# 2-(2-Methyl-5-nitro-1*H*-imidazol-1-yl)ethyl 2-bromo­benzoate

**DOI:** 10.1107/S1600536812012688

**Published:** 2012-03-28

**Authors:** Aurang Zeb, Sammer Yousuf, Fatima Z. Basha

**Affiliations:** a H.E.J. Research Institute of Chemistry, International Center for Chemical and Biological Sciences, University of Karachi 75270, Pakistan

## Abstract

In the title compound, C_13_H_12_BrN_3_O_4_, the dihedral angle between the benzene and imidazole rings is 30.6 (2)°. In the crystal, mol­ecules are linked into chains parallel to [001] by C—H⋯O hydrogen bonds. The crystal packing is further consolidated by π–π inter­actions [centroid–centroid distance = 3.482 (2) Å].

## Related literature

For background information and the crystal structure of the 4-flouro analogue of the title compound, see: Yousuf *et al.* (2012 ▶).
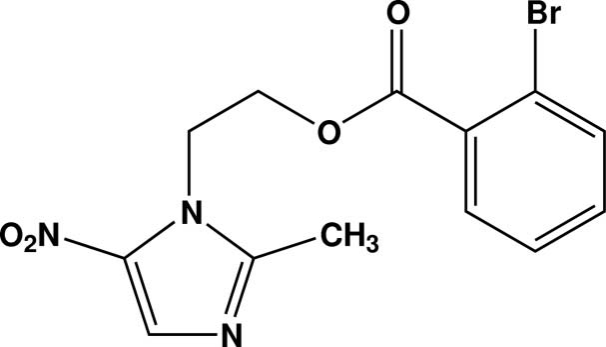


## Experimental

### 

#### Crystal data

C_13_H_12_BrN_3_O_4_
*M* *_r_* = 354.17
Monoclinic, 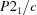

*a* = 14.554 (4) Å
*b* = 8.836 (2) Å
*c* = 11.563 (3) Å
β = 105.427 (6)°
*V* = 1433.3 (7) Å^3^
*Z* = 4
Mo *K*α radiation
μ = 2.89 mm^−1^
*T* = 273 K
0.33 × 0.20 × 0.19 mm

#### Data collection

Bruker SMART APEX CCD area-detector diffractometer
Absorption correction: multi-scan (*SADABS*; Bruker, 2000 ▶) *T*_min_ = 0.449, *T*_max_ = 0.610
8200 measured reflections
2601 independent reflections
1959 reflections with *I* > 2σ(*I*)
*R*_int_ = 0.024

#### Refinement

*R*[*F* ^2^ > 2σ(*F* ^2^)] = 0.042
*wR*(*F* ^2^) = 0.114
*S* = 1.04
2601 reflections
191 parameters
H-atom parameters constrained
Δρ_max_ = 0.68 e Å^−3^
Δρ_min_ = −0.51 e Å^−3^

### 

Data collection: *SMART* (Bruker, 2000 ▶); cell refinement: *SAINT* (Bruker, 2000 ▶); data reduction: *SAINT*; program(s) used to solve structure: *SHELXS97* (Sheldrick, 2008 ▶); program(s) used to refine structure: *SHELXL97* (Sheldrick, 2008 ▶); molecular graphics: *SHELXTL* (Sheldrick, 2008 ▶); software used to prepare material for publication: *SHELXTL*, *PARST* (Nardelli, 1995 ▶) and *PLATON* (Spek, 2009 ▶).

## Supplementary Material

Crystal structure: contains datablock(s) global, I. DOI: 10.1107/S1600536812012688/pv2520sup1.cif

Structure factors: contains datablock(s) I. DOI: 10.1107/S1600536812012688/pv2520Isup2.hkl

Supplementary material file. DOI: 10.1107/S1600536812012688/pv2520Isup3.cml

Additional supplementary materials:  crystallographic information; 3D view; checkCIF report

## Figures and Tables

**Table 1:** Hydrogen-bond geometry (Å, °)

*D*—H⋯*A*	*D*—H	H⋯*A*	*D*⋯*A*	*D*—H⋯*A*
C8—H8*A*⋯O4^i^	0.97	2.51	3.183 (4)	127

Symmetry code: (i) 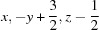.

**Fig. 1.**
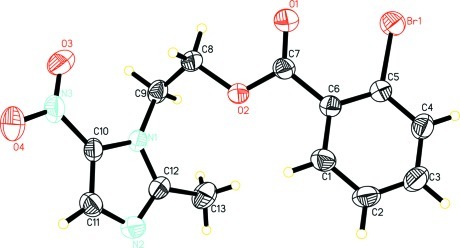
The molecular structure of the title compound with the atom numbering scheme. Displacement ellipsoids are drawn at the 30% probability level. H atoms are presented as small spheres of arbitrary radius.

**Fig. 2.**
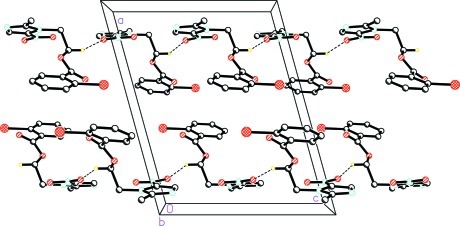
A view of the C—-H···O hydrogen bonds (dotted lines) in the crystal structure of the title compound. H atoms non-participating in hydrogen- bonding were omitted for clarity.

## supplementary crystallographic information

### Comment

The title compound is an ester derivative of a broad spectrum antibiotic, metronidazole, commonly known as flagyl. In continuation of our research (Yousuf *et al.*, 2012) we have now synthesized the title compound to evaluate its antiglycation potential. In this article, we report the synthesis and crystal structure of the title compound.

The title compound (Fig. 1) is structurally similar to its 4-flouro analogue (Yousuf *et al.*, 2012). The mean planes of the benzene (C1–C6) and imidazole (C10—C12/N1—N2) rings are inclined at 30.6 (2)° with respect to each other. In the crystal structure, the molecules are linked to form chains *via* C8—H8A···O4 intermolecular interactions along the *c*-axis (Fig. 2 and Tab. 1). The Crystal packing is further strengthened by a significant π–π interaction between centroids of imidazole rings lying about inversion centers (*Cg*···*Cg* distance = 3.482 (2) Å).

### Experimental

The synthesis of the title compounds was acheived by reacting metronidazole (171 mg, 1.0 mmole) with 2-bromobenzoic acid (1.2 equiv.) in the presence of dicyclohexylcarbodiimide (245 mg, 1.2 mmole) and 4-dimethylaminopyridine (0.35 mmole) in dichloromethane (10 ml) at room temperature for 40–45 h. The progress of the reaction was monitored by TLC. The reaction was quenched with 20 ml HCl (0.5 *M*) and then basified with sat. NaHCO_3_. It was extracted with dichloromethane and evaporated in vaccuo to obtain a crude product. The crude product was purified by using silica gel chromatography (EtOAc: hexane, 3.0: 7.0 to 7.0: 3.0) which afforded the title compound in 85% yield. Recrystallization by the slow evaporation of a dichloromethane solution of the title compound afforded pure crystals found suitable for single-crystal X-ray diffraction studies. All chemicals were purchased from Sigma-Aldrich.

### Refinement

H atoms on methyl, methylene and methine were positioned geometrically with C—H = 0.96, 0.97 and 0.93 Å respectively, and constrained to ride on their parent atoms with *U*_iso_(H)= 1.2*U*_eq_(CH and CH_2_) and 1.5*U*_eq_(CH_3_). A rotating group model was applied to the methyl groups.

### Crystal data

**Table:**

C_13_H_12_BrN_3_O_4_	*F*(000) = 712
*M**_r_* = 354.17	*D*_x_ = 1.641 Mg m^−^^3^
Monoclinic, *P*2_1_/*c*	Mo *K*α radiation, λ = 0.71073 Å
Hall symbol: -P 2ybc	Cell parameters from 2494 reflections
*a* = 14.554 (4) Å	θ = 2.7–24.1°
*b* = 8.836 (2) Å	µ = 2.89 mm^−^^1^
*c* = 11.563 (3) Å	*T* = 273 K
β = 105.427 (6)°	Block, colorles
*V* = 1433.3 (7) Å^3^	0.33 × 0.20 × 0.19 mm
*Z* = 4	

### Data collection

**Table:**

Bruker SMART APEX CCD area-detector diffractometer	2601 independent reflections
Radiation source: fine-focus sealed tube	1959 reflections with *I* > 2σ(*I*)
Graphite monochromator	*R*_int_ = 0.024
ω scans	θ_max_ = 25.5°, θ_min_ = 2.7°
Absorption correction: multi-scan (*SADABS*; Bruker, 2000)	*h* = −17→17
*T*_min_ = 0.449, *T*_max_ = 0.610	*k* = −10→10
8200 measured reflections	*l* = −14→13

### Refinement

**Table:**

Refinement on *F*^2^	Primary atom site location: structure-invariant direct methods
Least-squares matrix: full	Secondary atom site location: difference Fourier map
*R*[*F*^2^ > 2σ(*F*^2^)] = 0.042	Hydrogen site location: inferred from neighbouring sites
*wR*(*F*^2^) = 0.114	H-atom parameters constrained
*S* = 1.04	*w* = 1/[σ^2^(*F*_o_^2^) + (0.0598*P*)^2^ + 0.7664*P*] where *P* = (*F*_o_^2^ + 2*F*_c_^2^)/3
2601 reflections	(Δ/σ)_max_ < 0.001
191 parameters	Δρ_max_ = 0.68 e Å^−^^3^
0 restraints	Δρ_min_ = −0.51 e Å^−^^3^

### Special details

**Table:**

Geometry. All e.s.d.'s (except the e.s.d. in the dihedral angle between two l.s. planes) are estimated using the full covariance matrix. The cell e.s.d.'s are taken into account individually in the estimation of e.s.d.'s in distances, angles and torsion angles; correlations between e.s.d.'s in cell parameters are only used when they are defined by crystal symmetry. An approximate (isotropic) treatment of cell e.s.d.'s is used for estimating e.s.d.'s involving l.s. planes.
Refinement. Refinement of *F*^2^ against ALL reflections. The weighted *R*-factor *wR* and goodness of fit *S* are based on *F*^2^, conventional *R*-factors *R* are based on *F*, with *F* set to zero for negative *F*^2^. The threshold expression of *F*^2^ > σ(*F*^2^) is used only for calculating *R*-factors(gt) *etc*. and is not relevant to the choice of reflections for refinement. *R*-factors based on *F*^2^ are statistically about twice as large as those based on *F*, and *R*- factors based on ALL data will be even larger.

### Fractional atomic coordinates and isotropic or equivalent isotropic displacement parameters (Å^2^)

**Table:**

	*x*	*y*	*z*	*U*_iso_*/*U*_eq_	
Br1	0.39287 (3)	0.03857 (5)	0.11564 (3)	0.0748 (2)	
O1	0.3629 (3)	0.3331 (3)	0.2376 (3)	0.1003 (11)	
O2	0.25716 (17)	0.3109 (3)	0.3428 (2)	0.0606 (6)	
O3	0.1411 (3)	0.7613 (3)	0.4002 (3)	0.0981 (10)	
O4	0.1559 (3)	0.8059 (3)	0.5864 (3)	0.1087 (11)	
N1	0.11680 (18)	0.4509 (3)	0.4402 (2)	0.0471 (6)	
N2	0.1099 (2)	0.3517 (4)	0.6143 (3)	0.0667 (8)	
N3	0.1438 (2)	0.7198 (4)	0.5010 (3)	0.0696 (9)	
C1	0.3499 (3)	0.0609 (4)	0.4570 (3)	0.0623 (9)	
H1A	0.3271	0.1295	0.5038	0.075*	
C2	0.3779 (3)	−0.0807 (5)	0.5015 (4)	0.0747 (11)	
H2B	0.3749	−0.1072	0.5783	0.090*	
C3	0.4103 (3)	−0.1832 (5)	0.4319 (4)	0.0778 (12)	
H3A	0.4292	−0.2792	0.4620	0.093*	
C4	0.4151 (3)	−0.1450 (4)	0.3185 (4)	0.0664 (10)	
H4A	0.4367	−0.2151	0.2717	0.080*	
C5	0.3879 (2)	−0.0030 (4)	0.2746 (3)	0.0498 (8)	
C6	0.3552 (2)	0.1034 (4)	0.3431 (3)	0.0475 (7)	
C7	0.3277 (3)	0.2592 (4)	0.3011 (3)	0.0541 (8)	
C8	0.2178 (3)	0.4561 (4)	0.2996 (3)	0.0618 (9)	
H8A	0.2169	0.4689	0.2160	0.074*	
H8B	0.2556	0.5368	0.3459	0.074*	
C9	0.1188 (3)	0.4590 (4)	0.3138 (3)	0.0575 (9)	
H9A	0.0875	0.5514	0.2785	0.069*	
H9B	0.0832	0.3743	0.2704	0.069*	
C10	0.1311 (2)	0.5647 (3)	0.5247 (3)	0.0511 (8)	
C11	0.1268 (3)	0.5010 (4)	0.6297 (3)	0.0631 (9)	
H11A	0.1343	0.5528	0.7016	0.076*	
C12	0.1047 (2)	0.3241 (4)	0.4998 (3)	0.0555 (8)	
C13	0.0869 (3)	0.1719 (4)	0.4440 (4)	0.0826 (12)	
H13A	0.0630	0.1058	0.4952	0.124*	
H13B	0.1453	0.1316	0.4332	0.124*	
H13C	0.0408	0.1798	0.3675	0.124*	

### Atomic displacement parameters (Å^2^)

**Table:**

	*U*^11^	*U*^22^	*U*^33^	*U*^12^	*U*^13^	*U*^23^
Br1	0.1056 (4)	0.0702 (3)	0.0535 (3)	0.0046 (2)	0.0298 (2)	−0.00709 (18)
O1	0.134 (3)	0.0601 (16)	0.141 (3)	0.0125 (17)	0.096 (2)	0.0205 (18)
O2	0.0806 (16)	0.0593 (14)	0.0483 (13)	0.0208 (12)	0.0282 (12)	0.0138 (11)
O3	0.158 (3)	0.0530 (16)	0.095 (2)	−0.0046 (18)	0.054 (2)	0.0083 (15)
O4	0.169 (3)	0.0605 (17)	0.108 (3)	−0.011 (2)	0.056 (2)	−0.0356 (18)
N1	0.0498 (14)	0.0417 (14)	0.0480 (15)	0.0060 (11)	0.0097 (12)	−0.0016 (12)
N2	0.0730 (19)	0.068 (2)	0.0620 (19)	0.0077 (16)	0.0231 (16)	0.0130 (16)
N3	0.083 (2)	0.0497 (17)	0.081 (2)	0.0018 (15)	0.0311 (19)	−0.0153 (17)
C1	0.067 (2)	0.072 (2)	0.050 (2)	0.0040 (18)	0.0192 (17)	0.0049 (17)
C2	0.075 (3)	0.088 (3)	0.064 (2)	0.010 (2)	0.023 (2)	0.029 (2)
C3	0.074 (3)	0.063 (2)	0.097 (3)	0.010 (2)	0.025 (2)	0.028 (2)
C4	0.069 (2)	0.052 (2)	0.079 (3)	0.0061 (18)	0.020 (2)	0.0020 (19)
C5	0.0503 (18)	0.0488 (17)	0.0488 (19)	−0.0028 (14)	0.0106 (15)	−0.0015 (14)
C6	0.0488 (17)	0.0480 (16)	0.0456 (17)	−0.0023 (14)	0.0123 (14)	−0.0019 (14)
C7	0.070 (2)	0.0479 (17)	0.0491 (19)	−0.0027 (16)	0.0235 (17)	−0.0046 (15)
C8	0.088 (3)	0.054 (2)	0.047 (2)	0.0189 (18)	0.0245 (19)	0.0130 (16)
C9	0.074 (2)	0.0487 (19)	0.0434 (19)	0.0132 (17)	0.0044 (17)	0.0003 (15)
C10	0.0562 (19)	0.0445 (17)	0.054 (2)	0.0075 (14)	0.0159 (16)	−0.0058 (14)
C11	0.071 (2)	0.068 (2)	0.051 (2)	0.0093 (18)	0.0186 (18)	−0.0037 (17)
C12	0.0513 (19)	0.0473 (18)	0.066 (2)	0.0026 (15)	0.0128 (16)	0.0060 (17)
C13	0.095 (3)	0.050 (2)	0.102 (3)	−0.011 (2)	0.024 (3)	−0.002 (2)

### Geometric parameters (Å, º)

**Table:**

Br1—C5	1.895 (3)	C3—C4	1.374 (5)
O1—C7	1.195 (4)	C3—H3A	0.9300
O2—C7	1.326 (4)	C4—C5	1.372 (5)
O2—C8	1.439 (4)	C4—H4A	0.9300
O3—N3	1.213 (4)	C5—C6	1.393 (4)
O4—N3	1.221 (4)	C6—C7	1.479 (5)
N1—C12	1.351 (4)	C8—C9	1.494 (5)
N1—C10	1.379 (4)	C8—H8A	0.9700
N1—C9	1.472 (4)	C8—H8B	0.9700
N2—C12	1.328 (4)	C9—H9A	0.9700
N2—C11	1.345 (5)	C9—H9B	0.9700
N3—C10	1.420 (5)	C10—C11	1.355 (5)
C1—C2	1.372 (5)	C11—H11A	0.9300
C1—C6	1.391 (5)	C12—C13	1.485 (5)
C1—H1A	0.9300	C13—H13A	0.9600
C2—C3	1.376 (6)	C13—H13B	0.9600
C2—H2B	0.9300	C13—H13C	0.9600
			
C7—O2—C8	117.1 (3)	O2—C7—C6	111.7 (3)
C12—N1—C10	105.0 (3)	O2—C8—C9	106.5 (3)
C12—N1—C9	125.9 (3)	O2—C8—H8A	110.4
C10—N1—C9	129.0 (3)	C9—C8—H8A	110.4
C12—N2—C11	105.8 (3)	O2—C8—H8B	110.4
O3—N3—O4	123.4 (4)	C9—C8—H8B	110.4
O3—N3—C10	120.3 (3)	H8A—C8—H8B	108.6
O4—N3—C10	116.3 (4)	N1—C9—C8	112.5 (3)
C2—C1—C6	121.0 (3)	N1—C9—H9A	109.1
C2—C1—H1A	119.5	C8—C9—H9A	109.1
C6—C1—H1A	119.5	N1—C9—H9B	109.1
C1—C2—C3	119.6 (4)	C8—C9—H9B	109.1
C1—C2—H2B	120.2	H9A—C9—H9B	107.8
C3—C2—H2B	120.2	C11—C10—N1	107.4 (3)
C4—C3—C2	120.6 (4)	C11—C10—N3	127.8 (3)
C4—C3—H3A	119.7	N1—C10—N3	124.8 (3)
C2—C3—H3A	119.7	N2—C11—C10	109.8 (3)
C5—C4—C3	119.6 (4)	N2—C11—H11A	125.1
C5—C4—H4A	120.2	C10—C11—H11A	125.1
C3—C4—H4A	120.2	N2—C12—N1	112.1 (3)
C4—C5—C6	121.0 (3)	N2—C12—C13	123.8 (3)
C4—C5—Br1	117.1 (3)	N1—C12—C13	124.1 (3)
C6—C5—Br1	121.9 (2)	C12—C13—H13A	109.5
C1—C6—C5	118.0 (3)	C12—C13—H13B	109.5
C1—C6—C7	118.9 (3)	H13A—C13—H13B	109.5
C5—C6—C7	123.0 (3)	C12—C13—H13C	109.5
O1—C7—O2	122.4 (3)	H13A—C13—H13C	109.5
O1—C7—C6	125.9 (3)	H13B—C13—H13C	109.5
			
C6—C1—C2—C3	−0.9 (6)	C10—N1—C9—C8	80.5 (4)
C1—C2—C3—C4	0.1 (6)	O2—C8—C9—N1	64.2 (4)
C2—C3—C4—C5	0.5 (6)	C12—N1—C10—C11	−0.1 (4)
C3—C4—C5—C6	−0.2 (5)	C9—N1—C10—C11	−177.1 (3)
C3—C4—C5—Br1	−178.1 (3)	C12—N1—C10—N3	−177.7 (3)
C2—C1—C6—C5	1.2 (5)	C9—N1—C10—N3	5.3 (5)
C2—C1—C6—C7	−177.7 (3)	O3—N3—C10—C11	−176.0 (4)
C4—C5—C6—C1	−0.6 (5)	O4—N3—C10—C11	3.1 (6)
Br1—C5—C6—C1	177.1 (3)	O3—N3—C10—N1	1.1 (6)
C4—C5—C6—C7	178.2 (3)	O4—N3—C10—N1	−179.8 (3)
Br1—C5—C6—C7	−4.0 (5)	C12—N2—C11—C10	0.5 (4)
C8—O2—C7—O1	5.6 (5)	N1—C10—C11—N2	−0.2 (4)
C8—O2—C7—C6	−174.4 (3)	N3—C10—C11—N2	177.3 (3)
C1—C6—C7—O1	145.8 (4)	C11—N2—C12—N1	−0.6 (4)
C5—C6—C7—O1	−33.1 (6)	C11—N2—C12—C13	179.9 (3)
C1—C6—C7—O2	−34.2 (4)	C10—N1—C12—N2	0.5 (4)
C5—C6—C7—O2	147.0 (3)	C9—N1—C12—N2	177.6 (3)
C7—O2—C8—C9	156.0 (3)	C10—N1—C12—C13	179.9 (3)
C12—N1—C9—C8	−95.9 (4)	C9—N1—C12—C13	−3.0 (5)

### Hydrogen-bond geometry (Å, º)

**Table:**

*D*—H···*A*	*D*—H	H···*A*	*D*···*A*	*D*—H···*A*
C8—H8*A*···O4^i^	0.97	2.51	3.183 (4)	127

Symmetry code: (i) *x*, −*y*+3/2, *z*−1/2.

## References

[bb1] Bruker (2000). *SMART*, *SAINT* and *SADABS* Bruker AXS Inc., Madison, Wisconsin, USA.

[bb2] Nardelli, M. (1995). *J. Appl. Cryst.* **28**, 659.

[bb3] Sheldrick, G. M. (2008). *Acta Cryst* A**64**, 112–122. 10.1107/S0108767307043930 18156677

[bb4] Spek, A. L. (2009). *Acta Cryst.* D**65**, 148–155. 10.1107/S090744490804362X PMC2631630 19171970

[bb5] Yousuf, S., Zeb, A. & Basha, F. Z. (2012). *Acta Cryst.* E**68**, o952. 10.1107/S1600536812006319 PMC3343933 22590014

